# Potential of polyaniline modified clay nanocomposite as a selective decontamination adsorbent for Pb(II) ions from contaminated waters; kinetics and thermodynamic study

**DOI:** 10.1186/s40201-016-0261-z

**Published:** 2016-11-09

**Authors:** Somayeh Piri, Zahra Alikhani Zanjani, Farideh Piri, Abbasali Zamani, Mohamadreza Yaftian, Mehdi Davari

**Affiliations:** 1 Department of Chemistry, Faculty of Science, University of Zanjan, 45371-38791 Zanjan, Iran; 2 Department of Environmental Science, Faculty of Science, University of Zanjan, 45371-38791 Zanjan, Iran; 3 Iranian Research Organization for Science and Technology, Tehran, Iran

**Keywords:** Polyaniline, Clay, Nanocomposite, Nanolayers, Natural adsorbent, Water treatment, Heavy metals

## Abstract

**Background:**

Nowadays significant attention is to nanocomposite compounds in water cleaning. In this article the synthesis and characterization of conductive polyaniline/clay (PANI/clay) as a hybrid nanocomposite with extended chain conformation and its application for water purification are presented.

**Methods:**

Clay samples were obtained from the central plain of Abhar region, Abhar, Zanjan Province, Iran. Clay was dried and sieved before used as adsorbent. The conductive polyaniline was inflicted into the layers of clay to fabricate a hybrid material. The structural properties of the fabricated nanocomposite are studied by X-ray diffraction (XRD), Fourier transform infrared spectroscopy (FT-IR) and scanning electron microscope (SEM). The elimination process of Pb(II) and Cd(II) ions from synthetics aqueous phase on the surface of PANI/clay as adsorbent were evaluated in batch experiments. Flame atomic absorption instrument spectrophotometer was used for determination of the studied ions concentration. Consequence change of the pH and initial metal amount in aqueous solution, the procedure time and the used adsorbent dose as the effective parameters on the removal efficiency was investigated.

**Results:**

Surface characterization was exhibited that the clay layers were flaked in the hybrid nanocomposite. The results show that what happen when a nanocomposite polyaniline chain is inserted between the clay layers. The adsorption of ions confirmed a pH dependency procedure and a maximum removal value was seen at pH 5.0. The adsorption isotherm and the kinetics of the adsorption processes were described by Temkin model and pseudo-second-order equation. Time of procedure, pH and initial ion amount have a severe effect on adsorption efficiency of PANI/clay.

**Conclusions:**

By using suggested synthesise method, nano-composite as the adsorbent simply will be prepared. The prepared PANI/clay showed excellent adsorption capability for decontamination of Pb ions from contaminated water. Both of suggested synthesise and removal methods are affordable techniques.

## Background

Due to unique characteristics such as their interesting electrical and electrochemical properties, conducting polymers was used by many research groups worldwide. Among conducting polymers, polyaniline (PANI) has attracted considerable industrial interest and has been used in sensors fabrication [1, 2], electronic devices [3], batteries [4, 5], and as anti-corrosive additive inorganic coatings [6–8]. This wide range of applications motivates researchers to the development of PANI with improved characteristics. The process ability and some other properties of PANI could be enhanced by the synthesis of blending and composites compounds [9].

Polymers with two-dimensional nanomaterial’s structure, in particularly anisotropic platelet-like layered compounds such as layered silicates [10–12] have received more attention in recent years. Layered silicates platelets are exploited by a variety of methods and techniques [13–17]. Surface charge of these layers is permanent negative due to it is relocated by exchangeable inorganic cations same as Na^+^ and Ca^2+^. Silicate layers trend to hoard and form bundles. Therefore dispersing is an important need of individual Nano-platelets compounds within the polymer. The monomer molecules trend to penetration into the space between aggregate clay layers. Different levels of dispersion can be cratered based on the dispersion method used to fabricate the Nano-layer’s structure. The two ends of levels of dispersion are intercalated nanocomposite and exfoliated nanocomposite [18]. As an outcome, by controlling the amount of polymerized polymer in the clay layers at a low level, fully intercalated nanocomposite may be obtained. Clay nanocomposites can be used as a model for investigation on behavior of polymer confined in a two-dimensional space. Layered silicates/polymer nancomposites have been used for the sanitization of the wastewater due to their wide range of sources [19], readily available and much cheaper than adsorbents else.

Numerous methods such as solvent extraction [20], osmosis [21], chemical precipitation [22] and adsorption are famous and available methods for decontamination of heavy metals from wastewaters. Among these methods, adsorption [23] is preferable to have access to the goal. Among various effectual adsorbents such as activated carbon [24] and silica [25], clay is a suitable candidate for adsorption applications [26, 27]. This is due to the unique properties of clay [28–30]. Clay layered structures and ability to imprison water in the interlayer space raise the heavy metal adsorption and ion exchange. Therefore improvement of clay adsorption capacities by using different techniques is a favorable subject for researchers [28–30]. Same as clay other adsorbent was used in water refinement such as mainly polysaccharides such as chitosan [31], pistachio-nut shell ash [32], salvadora persica stem ash [33] and starch [34]. Low surface area and difficult separation from the water phase are disadvantages for natural polymers that decrease their use in field wastewater treatment applications.

Notable adsorption performance, low cost, wide availability and the presence of various functional groups on conducting polymeric composite materials are main cause that it has gained a distinctive attention [35]. Moreover, materials such as polyaniline have been used as profitable adsorbent for treatment aqueous solution of heavy metals ions. The different structural shape, special mechanism and environmental stability of PANI are mainly its reason [36–38].

Lead as a hazardous heavy metal is highly toxic to different types of living species on earth. Consuming contaminated waters with lead is a cause various types of serious diseases [39]. The suggested limit of lead ions is 10 μg L^−1^ in drinking water [40–42]. If 5 μg L^−1^ lead dissolved in drinking-water, the total intake of it can be calculated to range from 3.8 to 10 μg day^−1^ for an infant and an adult, respectively [41]. This is reported that by increasing the concentration of lead from the limits set by world health organization (WHO) and United States environmental protection agency (USEPA) (10 μg L^−1^), it impact the surrounding environment adversely and it can help to the outbreak of several diseases such as anemia, kidney damage and disorder in the nervous system [22].

In this report an easy, environmentally friend fabricating and economical method for synthesize nanocomposite from polyaniline and clay, via chemical grafting of PANI onto clay as a useful mineral adsorbent that is to find in nature abundantly, is demonstrated. The surface structure and morphology of the synthesized PANI/clay nanocomposite were studied by X-ray diffraction (XRD), Fourier transform infrared spectroscopy (FT-IR) and scanning electron microscope (SEM) techniques. Subsequently, the nanocomposites potency as decontamination agents was assessed in the removal of Pb(II) and Cd(II) ions in contaminated waters. The research of adsorption isotherms and kinetics of procedure were also done to understanding the adsorption behavior between the synthesized PANI/clay and the adsorbate ions. The whole of study was done in the summer of 2015 in the Environmental Science Research and Taghipour Dr. Laboratories, University of Zanjan, Zanjan-Iran.

## Method

### Materials and chemicals

Clay samples were obtained from the central Plain of Abhar region, Abhar, Zanjan Province, Iran. All used chemicals in this research with synthesis and analytical grade reagents were purchased from Merck or Fluka and were utilized in their initial form. Primal solutions of lead and cadmium ions with concentration 1000 mg L^−1^ were provided by dissolving a proper amount of corresponding nitrate salts in deionized water. Working solutions were obtained by appropriate dilution of the primal solutions with deionized water. For pH adjustments of solutions nitric acid and sodium hydroxide solutions were applied.

### Preparing of clay

Clay was first pretreated by the following procedure; at first, dried clay was sieved to 150 mm particle size then 30 g prepared clay was added into 300 mL concentrated sulfuric acid solution and the slurry mixtures was stirred for 1 week. Then, after separation of initial modified clay by filtration and it was washed thoroughly with distilled water until a time when the pH value of water filtrated was been 7.0. The crude product was dried before synthesis of the PANI/clay nanocomposite.

### Synthesis of the PANI/clay nanocomposite

The PANI/clay was synthesized via in situ chemical oxidative polymerization technique. In this manner that 2 g of acid modified clay was disorganized in water/ethanol mixture in a conical flask by sonication for 30 min. This procedure was done at room temperature. Then, 0.5 mL of aniline monomer was added and mixture again was sonicated for 20 min else for better diffusion of the monomer into the clay sheets. The monomer was polymerized by adding 0.42 g of ammonium persulphate and mixture stirring for 90 min else. The black mass was obtained as resulting nanocomposite and it was separated by filtration and washed with ethanol and distilled water repeatedly. The produced nanocomposite was air-dried at room temperature.

### Instruments

Field emission scanning electron micrographs (FESEM) was used for microscopy characterization morphological analyses in this propose the nanocomposite films were took by Mira 3-XMU system. Power X-ray diffraction patterns were performed for the PANI/clay nanocomposite on a Bruker D advance XRD meter between angle 2θ = 5-60° at 40 kV. Fourier transform infrared spectroscopy was carried out on a Bruker Vector 22 spectrophotometer. A flame atomic absorption spectrophotometer Varian 220A was used in quantitative analysis of metal ions concentration. A digital pH meter, Metrohm 780, was performed for pH adjustments.

### Adsorption measurements

Batch experiments in laboratory scale were selected to realize the effect of pH, time contacting and adsorbent dosage on behavior between PANI/clay adsorbent and studied ions. The solutions pH values were adjusted in the range 2–7 with HNO_3_/NaOH solutions (0.1 mol L^−1^). For investigation the adsorption behavior of the synthesized PANI/clay nanocomposite, 100 mg of it was put into 40 mL of 20 mg L^−1^ lead and cadmium ion solutions. This concentration was selected because that nerve conduction velocity is being appeared with increasing lead concentration from 20 mg L^−1^. Also lead ores comprise 20 mg Kg^−1^ of the earth^’^s crust [43]. The mixture of adsorbent and ions solution was mixed by using a magnetically stirring at laboratory temperature. After separation of the two mixed phases, the residual metal ion concentration in the aqueous phase was measured by FAAS. The amount of removed metal ions by per unit mass of used PANI/clay nanocomposites is computed by using eq. 1:

1 $$ {q}_e=\frac{\left({C}_0-{C}_e\right)\mathrm{V}}{m} $$

In this equation q_e_ note adsorbent adsorption capacity in the equilibrium time, C_0_ and C_e_ is the studied metal ion concentration (mg L^−1^) in zero and equilibrium time, respectively, m is the mass of the adsorbent (g), and V is the used volume solutions (L).

## Results and discussion

**Fig. 1:**
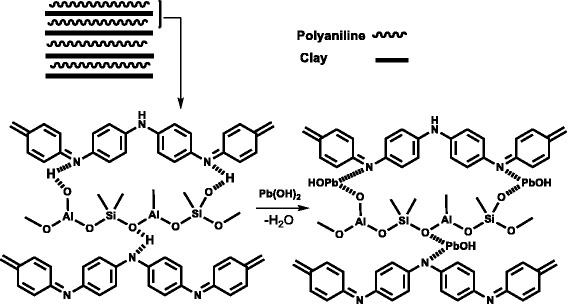
Mechanism of removal of Pb(II) by PANI/clay nanocomposites

It is very clear where nitrogen atoms exist in amine compounds due to the presence of electron in SP^3^ orbital of nitrogen can makes coordinate bond with positive charge of analytes. Figure 1 as a result mechanism introduce removal procedure of Pb(II) that may be explained with ion exchange between proton of amines in polyaniline or hydroxyl groups of clay nanocomposite with Pb(II) ions in water.

### Characterize analyses of PANI/clay nanocomposites

**Fig. 2:**
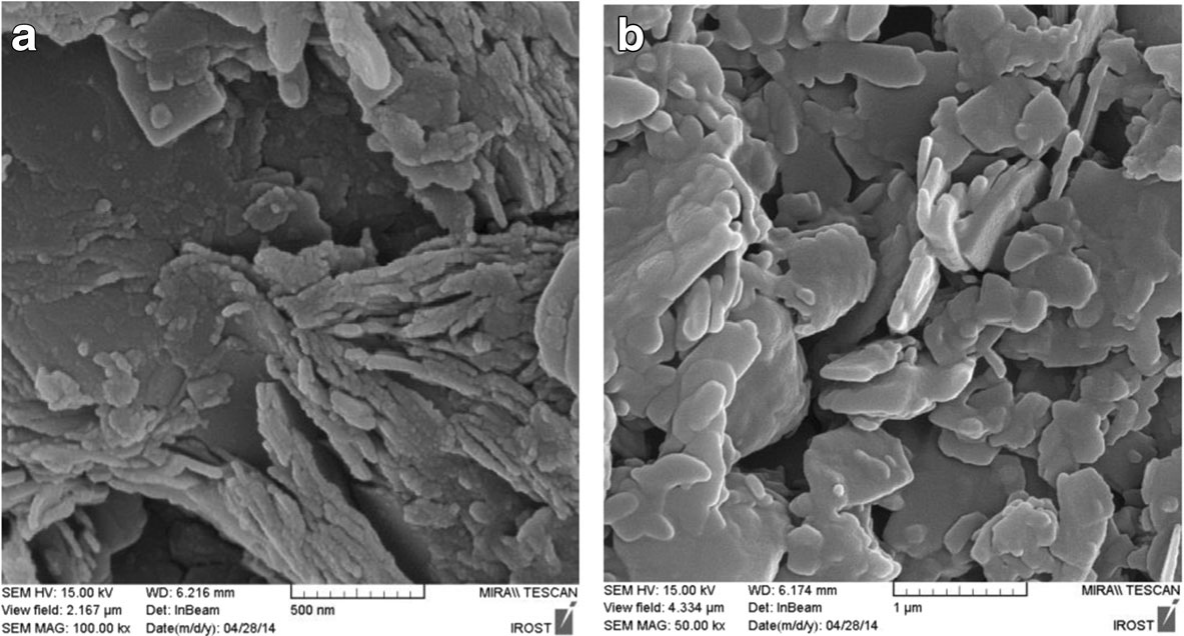
Scanning electron microscope images of clay (**a**) and PANI/claynanocomposites (**b**)

SEM technique was used to morphological analyses and characterization of the size and shape of the resulted PANI/clay nanocomposites (Fig. 2a). It shows the clay sheets as gray narrow plates have a very nice distribution in the synthesized nanocomposite (Fig. 2b). Also it confirmed that after modification of clay by PANI, the flaky clay structure coated by PANI and many individual platelets are seen in SEM images. This result reveals that PANI readily entered the layers of the clay and expanded and pushes it apart resulting in intercalated layered silicate PANI particles. Clay sheet thickness was near 40–50 nm.

**Fig. 3:**
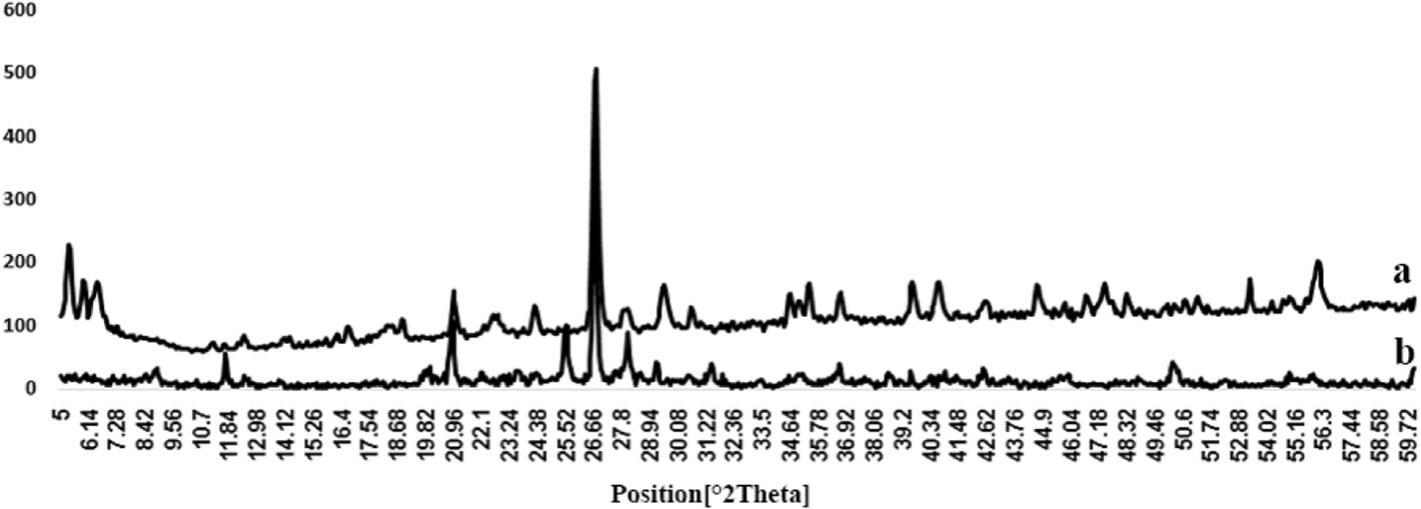
X-ray diffraction patterns of the clay **a** and PANI/clay **b** nanocomposites

The XRD pattern of the clay and PANI/clay nanocomposite in the 2θ range of about 5–60° are presented in Fig. 3. In the PANI/clay nanocomposite XRD image, the main peaks are similar to the clay particles, which confirmed that the crystalline structure of clay is nicely protected after the coating step under polymerization process. Due to the relatively thin layer and amorphous crystallinity of the PANI prepared under this polymerization method, no obvious diffraction peak for the PANI is detected. XRD Result for the clay represented in Table 1.

**Table 1:** Representative XRD analysis of clay

Ref. code	Score	Compound name	Scale factor	Chemical formula
01-086-1628	18	Quartz low	0.607	SiO_2_
00-013-0259	7	Montmorillonite-14A	0.194	Na_0.3_ (Al, Mg)_2_ Si_4_O_10_ (OH)_2_ !x H_2_O
00-003-0015	4	Montmorillonite (bentonite)	0.097	(Na, Ca)_0.3_ (Al, Mg)_2_ Si_4_O_10_ (OH)_2_!x H_2_O
00-029-0989	4	Merlinoite	0.087	K_5_Ca_2_(Al_9_Si_23_O_64_) !24 H_2_O
01-076-0885	2	Biotite 2 M1	54.925	KMg_2_Al_2_Si_3_O_11_(OH)
01-079-1343	0	Dolomite	0.092	CaMg(CO_3_)_2_
01-086-2339	1	Calcite	0.415	Ca(CO_3_)
00-006-0046	2	Gypsum	0.046	CaSO_4_ !2 H_2_O

**Fig. 4:**
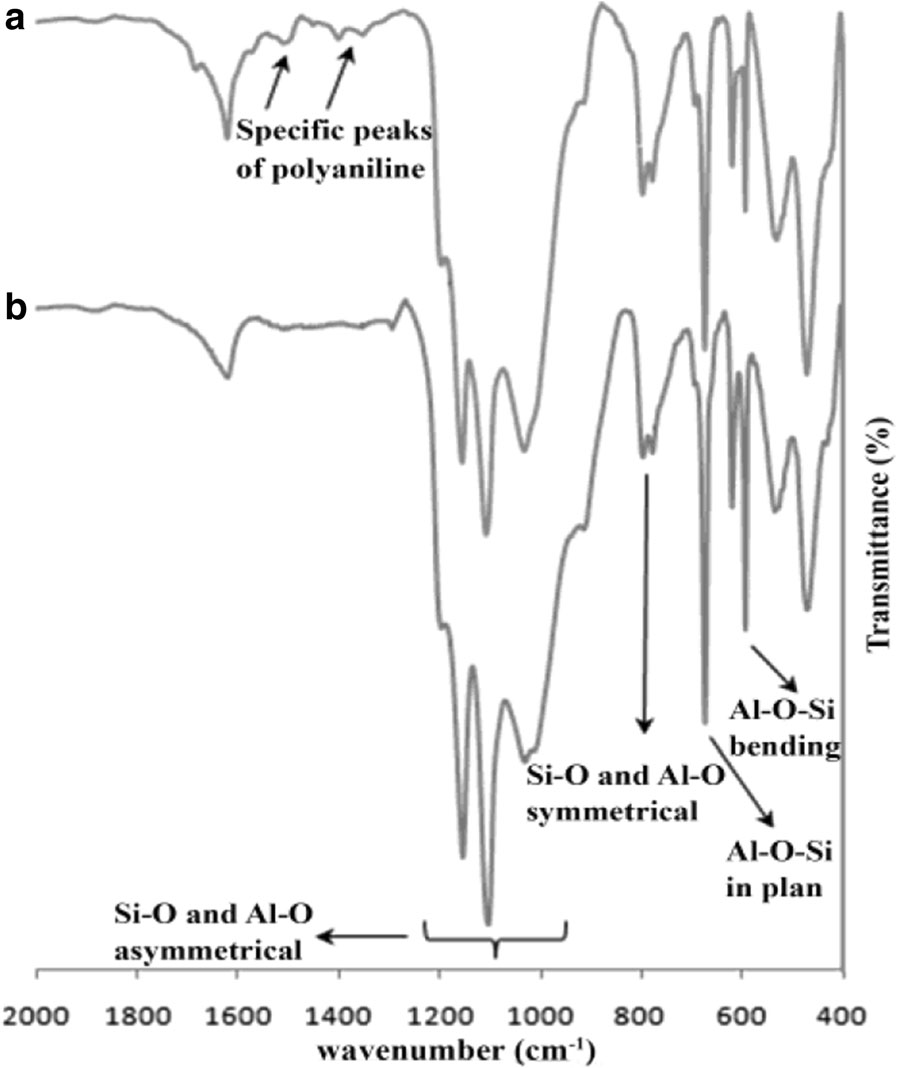
FTIR spectra in KBr pellets of PANI/clay (**a**) and clay (**b**)

Figure 4 exhibits the Fourier transform infrared spectroscopy spectra of the KBr pellet PANI/clay (a) and clay (b) specimens in the wavenumber of 2000–400 cm^−1^. In the PANI/clay specimens, the absorption bands at 1567 and 1489 cm^−1^ are appointed to the stretching vibration of Quinone and benzene rings of PANI compound, respectively. For both spectra of clay (b) and PANI/clay (a), the absorption peak at 1641 cm^−1^ is dependent to the H–O–H vibration in water bending, and the Si-O and Al-O asymmetrical stretching vibration was appeared at 1079 cm^−1^ and 1167 cm^−1^. Also, the appeared peaks at 798 cm^−1^ and 695 cm^−1^ were assigned to the Si-O and Al-O symmetrical stretching and bending vibrations, respectively. In the PANI/clay nanocomposite FT-IR spectra (a) the peaks correspond to Si-O and Al-O at 798 cm^−1^ are slightly shifted to higher asymmetric frequency which may be due to interactions like Vander Waal’s forces and hydrogen bonding.

### Application PANI/clay nanocomposite as adsorbent for removal of heavy metal ions

In order to assessment of adsorption capacity, the obtained synthesized PANI/clay were applied as an adsorbents for decontamination of Pb(II) and Cd(II) ions from polluted aqueous solutions. The effective parameters on the adsorption process such as pH of aqueous solution, contact time and sorbent dose is studied. Then the synthesized PANI/clay was used for treatment of real water samples that it is polluted with lead ions.

### Influence of working solution pH

**Fig. 5:**
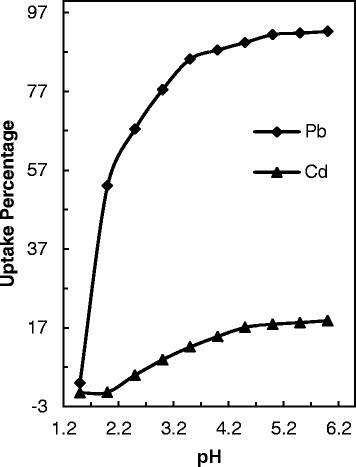
Pb(II) and Cd(II) ions removal with PANI/clay nanocomposite versus pH of solution

Due to effect on solubility of adsorbate, concentration of the studied ions on the adsorbent functional groups and degree of ionization and deformation of the adsorbate and adsorbent during reaction, the pH effect of a solution content adsorbate is an important study in adsorption studies [30]. Therefore, in the first step of this study the role of pH in the maximum removal of studied ions was examined over a pH range of 2.0–7.0. Figure 5 shows that the total removal percent of lead ions by PANI/clay increases with an increase in pH from 2.0 to 3.0 and the maximum removal around an initial pH = 4.0 can be seen. By direction initial pH of solution to basic range excited Pb-N bond formation between the reactive groups on the PANI/clay and Pb(II). Also in basic solutions, by releasing the protons from the imine groups, more activated binding sites are available for Pb(II) ions. As shown in Fig. 5, the high removal percent was seen at pH = 5–6. By increasing pH from 2 to 6, the Pb(II) adsorbed raised from 4.2 to 7.4 mg g^−1^.

At aqueous solution with pH < 6, the majority presented lead specie is Pb(II) form and the decontamination of Pb(II) is mainly done by sorption reaction. Therefore, the low removal Pb(II) ions at acidic solutions can be illustrated to the competition between H^+^ and Pb^2+^ ions on the activated surface sites of adsorbent [44, 45].

The same behavior for pH effect have been reported by Jiang et al., [30] in using modified kaolin as adsorbent for Pb(II) ions. It is shown highest adsorption was seen at final pH > 4 and increasing pH of aqueous solution increases amount of adsorbed ions [30]. Also comparison of both result confirmed that the capability of present studied adsorbent in lead removal is lower than modified kaolin.

Also surfactant emulsion membrane technology was used by Lende [22] for removal of Pb(II) from printed circuit board (PCB). In this study the pH of the filtered waste water was found to be around 5 and pH 4 is optimum amount in the removal of Pb(II) ions (initial concentration 150 mg L^−1^) with 82 % extraction [22]. Therefore the quantitative removal at pH 5–6 (in the present study) is good for decrease lead ions from PCB wastewater.

### Time dependency

**Fig. 6:**
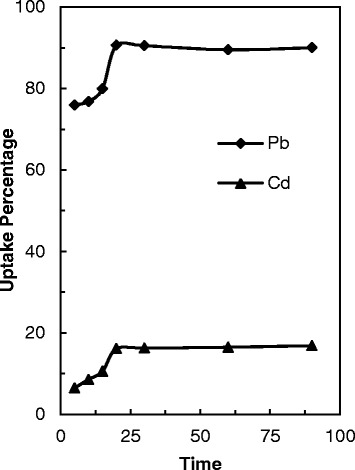
Dependency of contact time for adsorption studied ions onto PANI/clay nanocomposite

Equilibrium time as one other important parameters show the need time for removal in adsorption procedure. This is important in design procedure for pilot or industry scale. The contact time dependency of the removal procedure of Pb(II) ions by PANI/clay adsorbent is given in Fig. 6.

Figure 6 show that the removal of Pb(II) and Cd(II) ions by the used PANI/clay nanocomposite as adsorbents are a quick process, removal percent for both studied ions increase with the contact time, where over 90 % of lead ions removal was done within the first 20 min and equilibrium time is about 25 min. The reason of quick removal of Pb(II) ions at the initial times may be due to excess active sites on the uncovered surface of adsorbents. With increasing contact time and decreasing the active adsorption sites on PANI/clay nanocomposite as well as initial studied ion concentration, the adsorption became firstly slow and then fixed and steady curve can be seen. As another results in study of time dependency the maximal removal of Pb(II) is noticeable than removal of Cd(II). To complete the adsorption study versus contact time, the pseudo-first order and pseudo-second order kinetic models was used as the usefulness and famous models for study and determine of the kinetic parameters for adsorption procedure. By quickly covering of the active sites on the PANI/clay nanocomposite by Pb(II) ions the removal percent is dependent on the transported rate of the ions that penetrate from the bulk liquid phase to activated adsorption sites [30].

### Effect of dosage adsorbents

**Fig. 7:**
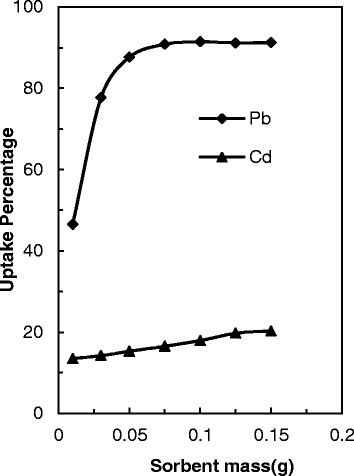
Effect of adsorbent amount on Pb(II) and Cd(II) ions onto PANI/clay nanocomposite

The removal of Pb(II) and Cd(II) ions versus the adsorbent dosage (0.01–0.15 g) at aqueous solution with initial lead concentrations of 100 mg L^−1^ and pH 5 is demonstrated at Fig. 7. This Figure shows that the removal of Pb(II) ions per gram of used PANI/clay nanocomposite sharply raises with increasing adsorbent amount from 0.01 to 0.05 g. At higher amount of PANI/clay nanocomposite by increasing the surface area and active sites higher removal of the lead ions was done. Further in removal of Cd(II), increasing adsorbent amount did not any significant effect. Above of 0.06 g of adsorbed equilibrium status can be seen between solid and solution phase. This optimum condition was kept constant for the study of all other parameters.

### Adsorption kinetics

Kinetic studies of adsorption procedure show data about the mechanism, which is useful for planning the practical process. In this study, the kinetics of adsorption of Pb(II), Cu(II) and Zn(II) on PANI/clay nanocomposite were studied by using the frequently used models, the Lagergren pseudo-first order model expressed in equation 2 and the pseudo-second order model expressed in equation 3 [38].

2 $$ ln\left({q}_e-{q}_t\right)= ln{q}_e-{K}_1t $$

3 $$ \frac{t}{q_t}=\frac{1}{k_2{q}_e^2}+\frac{t}{q_e} $$

Where q refer to the amount of analyte in mg g^−1^ and subscripts e and t show equilibrium and at any time, respectively and K_1_ (min^−1^) and K_2_ (g mg^−1^ min^−1^) in this equations denote the equilibrium rate constant corresponded to pseudo-first order and pseudo-second order adsorption, respectively.

A linear plot of log(q_e_- q_t_) versus t for this model was employed and the achieved R^2^ values for Pb(II) and Cd(II) ions are 0.977, 0.985, respectively.

**Fig. 8:**
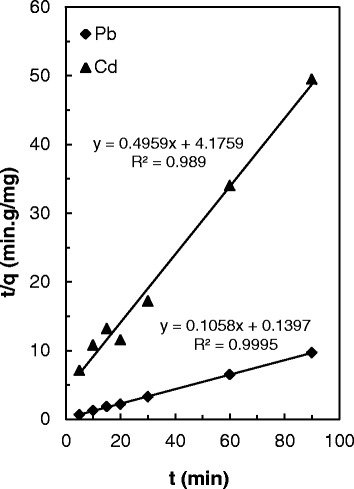
Pseudo-second order kinetic plot of Pb(II) and Cd(II) adsorption onto PANI/clay nanocomposite

The pseudo-second order adsorption can be obtained from the plot of t/q_t_ against t. The application of the model show that Linear plots of t/q_t_ versus t are obtained with R^2^ values of 0.989, for the Pb(II) and 0.999 for Cd(II) ions. This confirms that interaction between the studied ions and PANI/clay nanocomposite follow from the pseudo second-order mechanism (Fig. 8). It can be said that the rate limiting step is chemical interaction involving valence forces through sharing of electrons.

The result of studied mechanism indicates that removal of lead ions is subsequent to chemical reaction rather than physical-sorption. Also the quickly procedure in Pb(II) adsorption onto adsorbent show a chemical sorption which was done due to the strong electrostatic interaction between the negative charge on the PANI/clay nanocomposite surface and Pb(II) ions [30].

### Adsorption isotherms

The Langmuir, Freundlich and Temkin isotherm models was used for assessment of data of adsorption isotherm. These models describe the dependence between the adsorption amount of studied ions on the adsorbent surface and the equilibrium concentration of ions in the liquid phase.

The Langmuir isotherm and Freundlich equation used for monolayer and multilayer adsorption onto a surface, respectively. In the Langmuir isotherm identical active sites have finite number and Freundlich equation show heterogeneous surfaces [44]. Temkin isotherm model is a useful tool to estimate the adsorption heat due to correlation of adsorption heat of all molecules and temperature [46].

The linear equations of the Langmuir, Freundlich and Temkin isotherms can be expressed in the equations 4, 5 and 6, respectively:

4 $$ \frac{{\mathrm{C}}_{\mathrm{e}}}{{\mathrm{q}}_{\mathrm{e}}}=\frac{1}{{\mathrm{b}\ \mathrm{q}}_{\max }}+\frac{{\mathrm{C}}_{\mathrm{e}}}{{\mathrm{q}}_{\max }} $$

5 $$ \log {\mathrm{q}}_{\mathrm{e}}=\frac{1}{\mathrm{n}} \log {\mathrm{C}}_{\mathrm{e}}+\kern0.6em  \log {\mathrm{K}}_{\mathrm{f}} $$

6 $$ {\mathrm{q}}_e=\frac{\mathrm{RT}}{{\mathrm{b}}_{\mathrm{T}}} \ln {\mathrm{A}}_{\mathrm{T}}{\mathrm{C}}_{\mathrm{e}} $$

Where C show the equilibrium concentration (mg L^−1^), q_max_ (mg g^−1^) denote the maximum adsorption capacity, b (L mg^−1^) relates the energy of adsorption, K_f_ indicates relative adsorption capacity (mg^1−(1/n)^ L ^1/n^g^−1^) and n is an empirical parameter related to the intensity of adsorption. A_T_ is Temkin isotherm equilibrium binding constant (L g^−1^) and b_T_ is Temkin isotherm constant respectively.

The effective parameters of the studied isotherm models obtained from regression analysis of the experimental data and they are summarized in Table 2. According these reported result, the Temkin isotherm justified experimental data better than the Langmuir and Freundlich isotherm in the studied lead concentration range. In Temkin isotherm model, B parameter (equations 7) shows heat of sorption (J mol^−1^).

**Table 2:** The obtained parameters in study of procedure isotherms

Ions	Isotherms parameters								
	langmuir			Freundlich			Temkin		
	b(L mg^−1^)	q_max_(mg g^−1^)	R^2^	n	K_f_(mg g^−1^)	R^2^	A_T_ (L g^−1^)	b_T_ (kJ mol^−1^)	R^2^
Pb(II)	0.04	70.42	0.74	1.01	4.16	0.87	0.87	0.15	0.98
Cd(II)	0.05	0.12	0.96	0.04	0.00	0.78	0.05	0.02	0.53

7 $$ \mathrm{B}=\frac{\mathrm{RT}}{{\mathrm{b}}_{\mathrm{T}}} $$

The values A_T_ = 0.87 L g^−1^, R_2_ = 0.98 and B = 16.52 J mol^−1^were estimated From the Temkin plot. The heat of sorption indicates a physical adsorption process.

### Effect of initial metal ion concentration

Pb(II) ion concentration was set in the ranges of 10, 30, 40, 50, 100, 200 and 500 mg L^−1^ to determine of maximum quantity removal. The rising initial Pb(II) concentration caused an increasing in the Pb(II) removal by using PANI/clay nanocomposite (the results not shown). With increasing initial lead ion concentration, the amount of metal ion adsorbed raised due to increasing driving force of the adsorber towards the active sites on both the modified and unmodified adsorbents [30]. Due to the saturation of binding sites, at higher concentrations, more Pb(II) as the adsorbers was returned in to solution. Also when initial Pb(II) concentration in aqueous solution was 200 mg L^−1^, the empirical maximum adsorption capacity calculated that it was 9.6 mg g^−1^.

### Desorption studies

Due to metal ion recycling and recovery of adsorbant, desorption study is important stage in adsorption process. As the quantitative desorption of the adsorbed lead ions on the PANI/clay nanocomposite by distilled water was not successful, thus, hydrochloric, nitric and sulfuric acids were used to this end. HCl and HNO_3_ presents higher desorption capacity towards lead ions. More than 80 % of all the adsorbed studied ions were left adsorbent surface under the using 5 mL of HCl and HNO_3_ as stripping solutions (0.1 M).

### Application of procedure for real samples

In order to investigate the matrix effect on suggested procedure, the addition method, with an addition of lead and Cadmium ions to drinking water, river water and sea water as real samples was used. The real matrixes commonly decrease of adsorption efficiency due to present high amount of interfering agent. Obtained results of study matrix effect are presented in Table 3, it proved that the presence of interfering ions and other reagents commonly found in real water have negligible influence on removal of Pb(II) ion by using PANI/clay nanocomposite.

**Table 3:** Adsorption of Pb(II) and Cd(II) from the real water samples

Ions	Removal (%)		
	Drinking water	River water	Sea water
Pb(II)	90.7	91.2	91.7
Cd(II)	16.2	15.9	16.4

## Conclusions

Clay was found as a suitable substrate or support for coating of polyaniline. The results of nanocomposite characterization confirmed that the clay sheets were become layered in the prepared nanocomposite. The sorption capacity by modified sorbent was strongly dependent on contact time, pH, and initial ion concentration. The metal uptake was found to increase with pH. It was also found that the sorption of Pb(II) by polyaniline/clay appeared to follow the Temkin isotherm. Adsorption kinetics followed the pseudo-second-order model with very good correlation coefficients for Adsorption. In other hand Temkin is good isotherm model for studied process. The pseudo-second-order as kinetic model and Temkin as isotherm model confirm a companionship physical and chemical adsorption in studied removal process. In this concern, complete removal of contaminating lead ions was achieved in the real samples under investigations. It might be concluded that polyaniline modified clay nanocomposite are promising adsorption system in future water and wastewater treatment in order to remove lead ion. The present study highlights is the introducing new method in synthesize clay nanocomposite which have low price and first application of polyaniline modified clay nanocomposite as a sorbent for water treatment of lead ions. Comparison of the adsorption efficiency of polyaniline/clay and other materials is presented in Table 4.

**Table 4:** PANI/clay nanocomposite for lead removal against various reported adsorbents

Adsorbent	Maximum Adsorption Capacity (mg g^−1^)^a^	Adsorption isotherm	Adsorption Kinetic model	References
Polyaniline/clay	70.4	Temkin	pseudo-second order	Present study
Unmodified kaolinite clay	4.7	Langmuir	pseudo-second order	[30]
Modified kaolinite clay	32.2	Langmuir	pseudo-second order	[30]
polyaniline on multiwalled carbon nano-tubes	22.2	Langmuir	-	[37]
Peganum harmala seeds	90.0	Freundlich	pseudo-second order	[44]

^a^Calculated from Langmuir isotherm

## Abbreviations

FT-IR: Fourier transforms infrared spectroscopy
PANI: Polyaniline
PANI/clay: Polyaniline/Clay
SEM: Scanning electron microscope
USEPA: United States Environmental Protection Agency
WHO: World Health Organization
XRD: X-ray diffraction
